# A Mobile-based Virtual Reality Speech Rehabilitation App for Patients With Aphasia After Stroke: Development and Pilot Usability Study

**DOI:** 10.2196/30196

**Published:** 2022-04-07

**Authors:** Xiaofan Bu, Peter HF Ng, Ying Tong, Peter Q Chen, Rongrong Fan, Qingping Tang, Qinqin Cheng, Shuangshuang Li, Andy SK Cheng, Xiangyu Liu

**Affiliations:** 1 Nursing Teaching and Research Section Hunan Cancer Hospital/The Affiliated Cancer Hospital of Xiangya School of Medicine Central South University Changsha China; 2 Department of Computing The Hong Kong Polytechnic University Hong Kong China; 3 Department of Rehabilitation Brain Hospital of Hunan Province Hunan University of Chinese Medicine Changsha China; 4 Faculty of Medicine Nethersole School of Nursing Chinese University of Hong Kong Hong Kong China; 5 Department of Nursing Second Xiangya Hospital of Central South University Changsha China; 6 Department of Rehabilitation Sciences The Hong Kong Polytechnic University Hong Kong China; 7 Department of Health Service Center Hunan Cancer Hospital/The Affiliated Cancer Hospital of Xiangya School of Medicine Central South University Changsha China

**Keywords:** virtual reality, speech rehabilitation, stroke, app, Delphi

## Abstract

**Background:**

Stroke has the highest disability-adjusted life-years lost in any disease, and approximately one-third of the patients get aphasia. Computers and tablets are innovative and aid in intensive treatments in speech rehabilitation for patients with aphasia. However, mechanical training limits the help to patients.

**Objective:**

This study aims to provide a framework for an integrated virtual reality (VR) app to provide speech rehabilitation for patients with aphasia.

**Methods:**

The content was generated through an in-depth literature review and discussion with experienced rehabilitation physicians and occupational therapists. We then conducted a 2-round Delphi study with 15 experts from hospitals and universities to rate the content using a 5-point Likert scale. The app was developed by an interdisciplinary team involving VR, medical science of rehabilitation, and therapeutic rehabilitation. Pilot usability testing of this novel app was conducted among 5 patients with aphasia, 5 healthy volunteers, 5 medical staff, and 2 VR experts.

**Results:**

We designed 4 modules of speech rehabilitation: oral expression, auditory comprehension, cognition, and comprehensive application. Our VR-based interactive and intelligent app was developed to provide an alternative option for patients with aphasia. Pilot usability testing revealed user satisfaction with the app.

**Conclusions:**

This study designed and tested a novel VR-based app for speech rehabilitation specifically adapted to patients with aphasia. This will guide other studies to develop a similar program or intelligent system in a clinical setting.

## Introduction

### Background

Aphasia is an acquired language impairment following acquired brain injury (ABI) that affects some or all language modalities, including the expression and understanding of speech, reading, writing, and gestures [1]. ABI is a rapidly growing public health problem resulting from traumatic brain injury, stroke, hypoxic-ischemic encephalopathy after cardiac arrest, and brain tumors [2]. Stroke leads to the highest disability-adjusted life-year loss in any disease, with over 2 million new cases annually in China [3]. It is estimated that there are 1.1 million stroke-related deaths in China per year, and this number is increasing [3]. Globally, the number of stroke deaths is projected to rise to 7.8 million by 2030 [4].

Approximately one-third of stroke patients experience aphasia [1]. Patients with aphasia have a higher risk of not returning to work than those without aphasia [5]. It is likely that an individual's inability to reenter the workforce poststroke is due to the presence of aphasia [5]. The incidence of stroke in younger patients was considerably lower than that in the older cohorts; however, it remains on the rise [6], and rehabilitation needs are worthy of attention [5]. It has been reported that 94% of individuals are diagnosed with cognitive or communicative deficits. However, only 45% were referred for speech-language pathology services [7]. Computers and tablets have proposed innovative and intensive treatments for patients with aphasia in language rehabilitation [8-10]. Naming abilities are improved in patients who receive training, whereas no significant improvements have been shown in verbal communication skills [8,10]. A review concluded that many apps identified from the Google Play Store, Apple App Store, and web searches are available to adults with communication disorders for speech-language therapy. However, few have been designed to specifically meet this vulnerable population's engagement, functionality, aesthetics, and information quality [11].

Mechanical training limits the help to patients. Communication activity in real-world settings is more effective for patients in improving their communicative ability [1]. Neither smartphones nor computers can make a patient feel like they are in a real-life environment. The development of virtual reality (VR) technology has created interactive computer-generated worlds through visual, listening, and touch simulations. This makes patients more enthusiastic and more willing to speak. Patients will be much more easily accessible to dangerous, expensive, uncontrollable, and inaccessible environments in VR than in hospitals and will be able to try out new therapeutic strategies [12]. It has been identified that speech treatment based on VR is effective. The novel VR platform, EVA Park, is an online virtual island that contains various simulated locations, including houses, cafés, restaurants, health centers, hair salons, tropical bars, and discos [13]. It is designed to enable patients with aphasia to communicate successfully with 1 or more conversation partners via speech using a headset and microphone in real time [13]. Mirror neuron rehabilitation training software combined with a 4-channel VR panoramic helmet and Z-channel independent training equipment, including training contents of nouns, verbs, phrases, and sentence listening and reading, is effective for patients with Broca aphasia [14]. Additionally, due to the VR system almost completely shielding interference from the outside world, patients focus more on speech treatment [14]. However, these systems are semi-immersive VR environments that require partners and the help of a therapist.

### Objectives

This study aims to provide a framework for an integrated VR app and perform a preliminary test of its usability and safety. Few apps are explicitly designed to meet the requirements of engagement, functionality, aesthetics, and information quality [11]. Therefore, we intended to add interactive and engaging elements by providing patients with opportunities to interact with a virtual environment and practice speaking in real-life scenarios.

## Methods

### Phase I

#### Designing the Contents of VR-Based Language Rehabilitation: a Delphi Study

**Figure 1:**
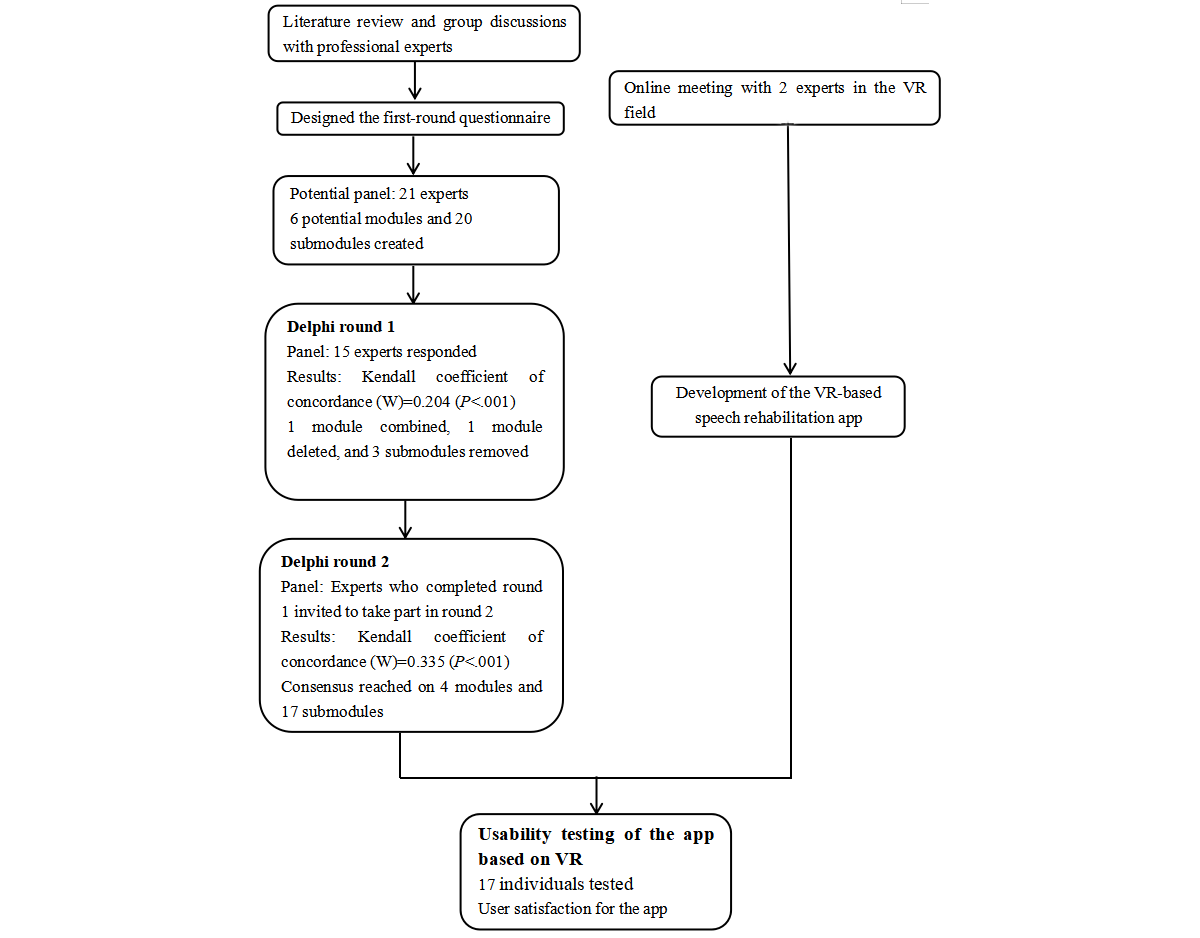
A 2-round Delphi study and development of the app. VR: virtual reality.

For the development of speech rehabilitation content based on VR for patients with aphasia after stroke, a 2-round Delphi study was conducted (Figure 1). The Delphi technique is used to obtain the most reliable consensus among a panel of experts using a series of questionnaires [15,16]. Experts can modify, add, or delete content, as appropriate [17]. We listed the module name, submodule name, specific contents, and a short description of the training modes during each Delphi round. Between 2 rounds, we revised the content based on expert feedback. In addition to written descriptions, the drafts included pictures and visual images.

#### Ethics Approval

The Ethics Review Committee of Nursing and Behavioral Medicine Research, School of Nursing, Central South University, approved the study (E202118; approval date: 21 April 2021).

#### Procedures and Participants

Before round 1 of the Delphi study, we conducted a literature review using the search terms “aphasia,” “speech,” “speech treatment,” “language therapy,” “communication,” and “rehabilitation” via the Wan Fang Database, Chinese National Knowledge Infrastructure (CNKI), PubMed, and Web of Science in September and October 2020 to identify available content and modes related to language rehabilitation. We developed the content by learning from a model of cognitive communication competence [7]. It includes 7 functioning domains: individual, contextual or environmental, cognitive, communication, physical/sensory, emotional/psychosocial, and communication competence. This model provides evidence that spans the fields of speech-language pathology, psychology, neuroscience, rehabilitation, and education, and concerns the complex interplay between cognitive, communicative, emotional, and physical factors. Rehabilitation platforms, such as Constant Therapy [18] and Aphasia Therapy Online [19], were retrieved. In addition, we learned about existing computer- or tablet-based speech rehabilitation systems.

In November 2020, we held group discussions with experienced rehabilitation physicians and occupational therapists at the Brain Hospital of Hunan Province and the Hunan Cancer Hospital to determine the relationship between brain regions and language production, the rehabilitation process of speech, and their opinions about current speech treatment. In December 2020, we held an online meeting with 2 experts in the Department of Computing at the Hong Kong Polytechnic University to generate ideas about potential content based on VR. Based on the information collected, we developed an initial draft of 20 submodules in 6 modules. We designed a questionnaire for experts to review and use a 5-point Likert scale to evaluate which part should be included. Each piece was described with a name, specific content, and a short description of the training modes.

The panel consisted of experts with both theory-based and practice-based backgrounds to obtain a variety of insights from researchers. Experts with a theory-based background all published papers in the speech treatment or rehabilitation fields. Experts with a practice-based background were selected based on their practical experience and publications. Additionally, we approached our network and asked responding experts to provide the names of essential experts in this field, such as a snowball sampling technique. The experts who participated in the first Delphi round were also approached in the second round. In total, 21 experts were invited by email, WeChat, or an in-person meeting to participate in the 2 rounds of the Delphi study. It contained detailed information about the goal, study procedure, first-round questionnaire, experts’ demographic information, and their judgment and familiarity with the corresponding field. Of the 21 experts, 15 (71.4% response rate) responded to the invitation.

#### First Round

##### Questionnaire

The first-round questionnaire was composed based on the contents identified by the literature review and group discussions. This resulted in a questionnaire consisting of 6 structured modules and 20 submodules. Experts were invited to rate these contents on a 5-point Likert scale ranging from 1 (not at all important) to 5 (extremely important). They were asked to modify, add, or delete content, as appropriate, and to provide suggestions they supposed were reasonable. The experts’ demographic information, as well as their judgment and familiarity with the corresponding field, was required. We reminded those who did not complete the survey following the initial invitation at 2-3-week intervals by WeChat or email.

##### Data Analysis

Microsoft Excel 2003 and IBM SPSS Statistics (version 26.0) were used for data analysis and processing. The responses of the experts were independently analyzed by 2 researchers, and the data were double-entered to minimize typing errors. We listed and combined similar recommendations. The mean, SD, and coefficient of variation (CV) were calculated. The CV is defined as the SD divided by the mean used to describe the relative dispersion degree of the items' importance evaluation by experts [20,21]. A lower CV value represents a higher degree of coordination among the experts' opinions [21]. After calculating the mean (SD) and CV that the contents should include, we removed all capabilities for which the mean score was less than 4.0 and the CV was over 0.2. Consensus among the experts was evaluated using the Kendall coefficient W test. It refers to the level of intraexpert agreement for all indicators [22]. Statistical significance was considered at a 2-tailed *P* value of <.05 [21]. Cs represents the experts' familiarity with the research field, and Ca represents the judgment criteria the experts are based on [21]. The defined values are listed in Tables 1 and 2. Cr represents the authority coefficient of experts, which is the mean of the sum of familiar Cs and Ca [23]. We then produced the round 2 questionnaire. Complete data were collected from 15 (71.4%) of the 21 panel members, and the results were used to revise the contents and establish a second questionnaire for round 2 of the Delphi exercise.

**Table 1:** The value of judgment (relevance) criteria.

Criterion	Influence degree		
	Great	Medium	Small
Theory analysis	0.5	0.4	0.3
Working experience	0.3	0.2	0.1
Referring to literatures	0.15	0.1	0.05
Self-intuition	0.05	0.1	0.15

**Table 2:** The degree of familiarity with content.

Familiarity degree	Very familiar	Familiar	General	Unfamiliar	Very unfamiliar
Self-evaluation	0.9	0.7	0.5	0.3	0.1

#### Second Round

##### Questionnaire

In February 2021, we held an online meeting with 2 experts in the VR field to discuss the content based on VR before the second round. Experts who completed the first-round questionnaire were invited to participate in round 2. Again, we reminded those who did not complete the survey following the initial invitation at 2-3-week intervals by WeChat or email. The experts rated each revised section again. They were asked to rate how much they agreed that each element could be used and to comment on each part. Additional advice about the content that they assumed reasonable was required.

##### Data Analysis

Analysis following round 2 aimed to identify any consensus on the contents and determine whether an additional round was needed. Microsoft Excel 2003 and IBM SPSS Statistics (version 26.0) were used for data analysis and processing. We predefined a mean score of no less than 4, a CV no more than 0.2, and a 2-tailed *P* value of the Kendall coefficient W test of no more than .05 among experts that the contents should be included. The mean, SD, and CV values were calculated for each part. The Delphi survey was completed when all the items in the questionnaire met the aforementioned criteria.

### Phase II

#### Development of the VR-Based Speech Rehabilitation App

In November 2020 and February 2021, we held online meetings with 2 experts in the VR field to discuss this project. A variety of devices and components can deliver a VR experience, with the main categories including smartphone VR headsets, tethered personal computer (PC)-based VR headsets (eg, HTC Vive), or stand-alone VR headsets (eg, Oculus Quest). HTC Vive and Oculus Quest create a more immersive experience and provide the user with a stronger sense of presence than smartphone VR owing to resolution, frame rate, and sufficient input mechanisms [24]. To provide an alternative option for patients under significant financial pressure, we chose to use smartphone VR and developed a mobile-based VR speech rehabilitation app. Smartphone VR headsets delivered a VR experience through a smartphone fitted on a headset, which was as simple as the original Google Cardboard. These types of VR apps are affordable and cost-effective [24]. Moreover, patients can also use existing smartphones.

**Figure 2:**
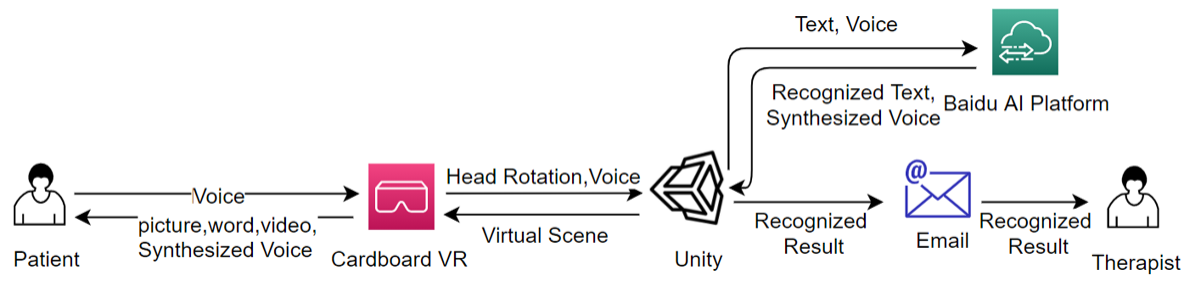
Detailed VR-based aphasia therapy process. AI: artificial intelligence; VR: virtual reality.

The VR scene was deployed on mobile phones using the Google Cardboard VR plug-in. Combining Google Cardboard equipment and mobile phones makes it a portable and cheap VR equipment that most patients can afford. The software can be used to receive language rehabilitation whenever and wherever possible. The entire VR scene was developed and constructed using the Unity game engine, and Adobe Photoshop was used for 2D assets. Unity is a bridge connecting artificial intelligence (AI) platforms, VR scenes, and back-end data storage. It uploads the designed text content to the Baidu AI platform and obtains a synthesized voice. The synthesized voice is used in the VR scene as a voice instruction to guide the user. Cardboard VR delivers pictures, words, videos, and synthesized voices to patients that substitute therapists in traditional treatment methods. The patients will see the designed questions and relevant materials in the VR scene and will record their voice answers using a mobile phone microphone or register their head rotation as an input for selecting answers in multiselection questions. Unity would then record their responses according to their head rotation or send the recorded voice to the Baidu AI platform to get the recognized text back. The results will be stored in an Excel sheet and sent to the therapist's email address with the patient’s name. Figure 2 elaborates on the detailed VR-based aphasia therapy process.

#### Pilot Application and Usability Testing of the App

We conducted feasibility exercises of the app on 5 patients with aphasia, 5 healthy volunteers, 5 medical staff, and 2 VR experts between 25 and 65 years of age (mean 39.53 years, SD 16.54 years). All 5 patients with aphasia were hospitalized for speech rehabilitation for 2 weeks, and all of them had ischemic stroke. The aphasia quotient (AQ) of the Chinese version of the Western Aphasia Battery ranged from 58 to 75 after 2 weeks of face-to-face training. This feasibility exercise allowed us to better understand and potentially reduce the likelihood of adverse effects in patients with aphasia who may have been more sensitive to sensory effects (eg, motion sickness and discomfort). Interviews were the measurements used in the experiments. The outline of interview questions after the participants’ experience with the mobile-based VR speech rehabilitation app is as follows: (1) What groups do you think this mobile-based VR speech rehabilitation app is suitable for? (2) How does the mobile-based VR speech rehabilitation app differ from face-to-face language rehabilitation? (3) What would encourage or hinder app adoption after using the mobile-based VR speech rehabilitation app? (4) What is your overall rating of the mobile-based VR speech rehabilitation app? All interviews were recorded using a digital voice recorder and transcribed verbatim within 24 hours. The researcher explored participants’ feelings about the app by asking questions in a private environment, and they were encouraged to put forward new questions and content.

## Results

### Results of the Delphi Study

#### Expert Panel

Table 3 lists the main characteristics of the 15 (71.4%) of 21 experts who participated in the first and second Delphi rounds. The 15 experts’ academic background included the medical science of rehabilitation or rehabilitation therapy. Of them, 14 (93.3%) were from 3A hospitals, 5 (33.3%) of them took a position in the university, and 2 (13.3%) of them were professors. Detailed demographic characteristics of the Delphi panel are presented in Table 3. The reliability of the experts is presented in Table 4. The mean value of the expert authority coefficient (Cr) was 0.84. The authority of the experts was high; thus, the results of this study are trustworthy.

**Table 3:** Characterization of experts participating in the Delphi panel (N=15).

Characteristic		Participants, n (%)
**Gender**		
	Male	2 (13.3)
	Female	13 (86.7)
**Age (years)**		
	<35	6 (40.0)
	≥35	9 (60.0)
**Education**		
	University diploma	9 (60.0)
	Master’s degree	4 (26.7)
	Doctoral degree	2 (13.3)
**Academic background**		
	Medical science of rehabilitation	5 (33.3)
	Rehabilitation Therapeutic	10 (66.7)
**Working experience (years)**		
	5-9	5 (33.3)
	10-14	5 (33.3)
	≥15	5 (33.3)
**Main area of the work role**		
	Clinical	10 (66.7)
	Clinical and research	5 (33.3)
**Professional title**		
	Intermediate	12 (80.0)
	Deputy senior	1 (6.7)
	Senior	2 (13.3)

**Table 4:** Reliability of experts (Ca^a^, Cs^b^, and Cr^c^).

Expert number	Criterion score						
	Theoretical analysis	Working experience	Referring to literature	Self-intuition	Ca	Cs	Cr
1	0.4	0.3	0.1	0.1	0.9	0.7	0.8
2	0.5	0.3	0.1	0.1	1	0.7	0.85
3	0.5	0.3	0.05	0.05	0.9	0.5	0.7
4	0.4	0.3	0.15	0.1	0.95	0.9	0.925
5	0.5	0.3	0.1	0.05	0.95	0.9	0.925
6	0.4	0.3	0.1	0.05	0.85	0.9	0.875
7	0.4	0.3	0.15	0.1	0.95	0.7	0.825
8	0.4	0.3	0.15	0.1	0.95	0.7	0.825
9	0.4	0.3	0.1	0.1	0.9	0.7	0.8
10	0.4	0.3	0.1	0.1	0.9	0.9	0.9
11	0.4	0.2	0.05	0.1	0.75	0.7	0.725
12	0.5	0.2	0.05	0.1	0.85	0.7	0.775
13	0.4	0.3	0.1	0.1	0.9	0.9	0.9
14	0.4	0.3	0.1	0.1	0.9	0.9	0.9
15	0.5	0.3	0.15	0.1	1.05	0.9	0.975

^a^Ca: judgment criteria the experts are based on.

^b^Cs: experts' familiarity with the research field.

^c^Cr: authority coefficient of experts.

#### First Round

The Kendall coefficient of concordance (W) was 0.204 (*P*<.001). Of the 6 modules and 20 submodules rated by the panel members in the first round (Table 5), the CV of 2 submodules (syllabic and naming with verbs, nouns, and adjectives) and the CV of the arithmetic therapy module that did not meet the criteria were removed (Table 6). In addition, 2 experts recommended to delete the arithmetic therapy module. Pictures and videos describing daily life were added based on the recommendations of 3 panel members. In addition, naming therapy was combined with oral expression therapy based on the advice of 2 panel members. Furthermore, 2 panel members suggested that single characters could be deleted because they are not as easy to express as phrases, so we deleted those. Some respondents suggested additional executive functioning, reading, and writing training. However, this app is meant to improve patients' oral expression abilities, so reading and writing are beyond our consideration, in particular, executive functioning and writing. Due to smartphone VR and technological limitations, we were unable to add writing training to VR. The revised content contained 4 modules and 17 submodules.

**Table 5:** Modules in round 1.

Module	Task
1	Oral expression therapy
2	Auditory comprehension therapy
3	Cognition therapy
4	Naming therapy
5	Arithmetic therapy
6	Comprehensive application

**Table 6:** Scores^a^ in round 1.

Tasks		Mean (SD)	CV^b^
**Oral expression therapy**			
	Read and repeat task: syllabic	4.13 (0.99)	0.24^c^
	Read and repeat task: single characters, including the 150 most commonly used Chinese characters	4.60 (0.91)	0.20^d^
	Read and repeat task: some phrases, including categories of digital, fruit, animals, vegetables, transportation, kitchen supplies, daily necessities, body parts, food, address, location, and sports	5 (0)	0.00
	Read and repeat task: sentences, including proverbs and daily expressions	4.87 (0.35)	0.07
	Answering some questions related to everyday life	4.80 (0.56)	0.12
**Auditory comprehension therapy**			
	Listening and matching: included digital, color, pictures, and words	4.93 (0.26)	0.05
	Yes-no questions	4.93 (0.26)	0.05
	Listening to the passage and answering questions	4.73 (0.46)	0.10
**Cognition therapy**			
	Attention training	4.27 (0.80)	0.19
	Memory training	4.13 (0.74)	0.18
	Reasoning and problem solving	4.47 (0.83)	0.19
**Naming therapy**			
	Picture naming	4.93 (0.26)	0.05
	Naming with verbs, nouns, and adjectives	4.33 (0.90)	0.21^c^
	Naming a list of items with the exact nature	4.93 (0.26)	0.05
**Arithmetic therapy**			
	Addition, subtraction, multiplication, and division	4.27 (1.16)	0.27^c^
**Comprehensive application**			
	Supermarket task: A computer-generated virtual customer purchases goods. The patient has to pick items, count the total amount of money that the virtual customer has selected, and return the change.	4.80 (0.56)	0.12
	Interview task: The patient plays the role of a job seeker. A computer-generated virtual interviewer asks questions about the patient’s personal information (eg, name, gender, age, nationality, education level, birth date, height, weight, specialty, address, and family members) and assesses their language skills by reading text and describing pictures.	4.73 (0.59)	0.13
	Bedroom task: The patient plays the role of a mother. By communicating with the computer-generated virtual daughter, the patient selects clothes for the virtual daughter according to the weather conditions and discusses breakfast and what to choose for dinner and the kind of transportation to use.	4.80 (0.56)	0.12
	Ordering task: The patient acts like a customer to order in a virtual restaurant; communicates with a virtual server synthesized by the computer; requests a certain number of dishes, desserts, cakes, and drinks; and completes the payment.	4.80 (0.41)	0.09
	Park task: By buying tickets, paying, and asking for directions in the virtual ticket office, together with a computer-generated virtual friend, the patient enters a virtual park. The patient and the virtual friend communicate with each other about the scene.	4.80 (0.41)	0.09

^a^Likert scale: 1, not important; 2, somewhat important; 3, moderately important; 4 = important; and 5, very important.

^b^CV: coefficient of variation.

^c^CV>0.2.

^d^Deleted on the recommendations of the 2 panel members.

#### Second Round

**Figure 3:**
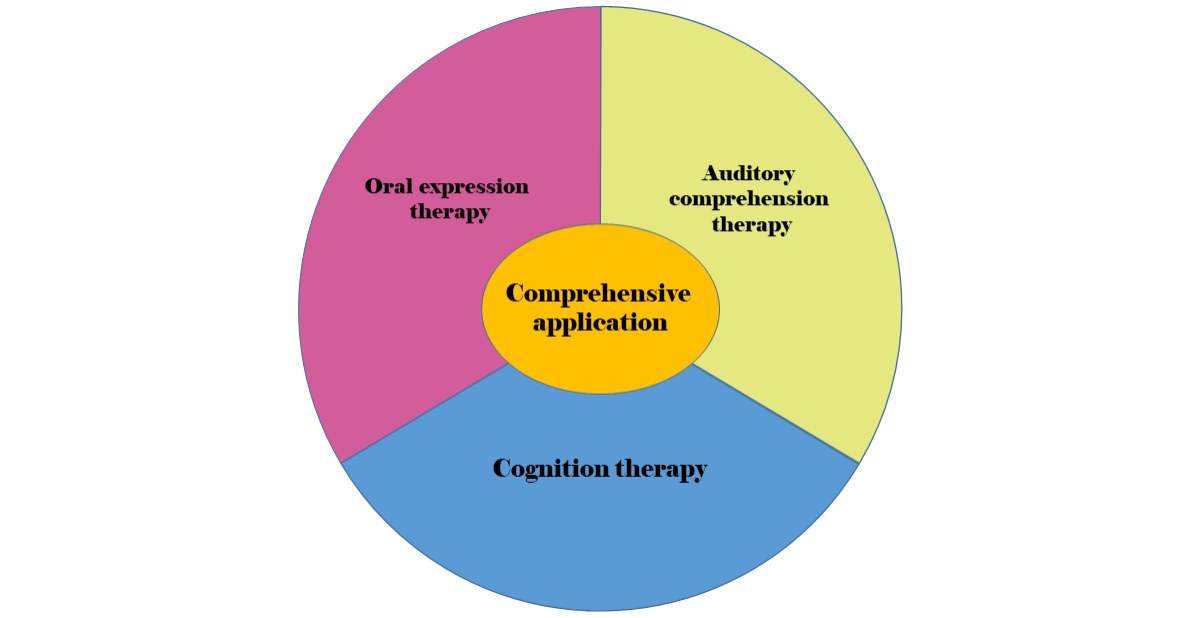
Consensus on 4 modules.

The results of the second round are presented in Tables 7 and 8 and Figure 3. All mean scores for importance were above 4.00, and the CVs were less than 0.20. The Kendall coefficient of concordance (W) was 0.335 (*P*<.001). Finally, a consensus was reached on 4 modules and 17 submodules. One expert suggested that patients should complete answering within the allotted time. Some respondents suggested additional executive functioning, reading, and writing training. However, this app is meant to improve patients' oral expression abilities, so reading and writing are beyond our consideration. The revised content contained 4 modules and 17 submodules.

**Table 7:** Modules in round 2.

Module	Task
1	Oral expression therapy
2	Auditory comprehension therapy
3	Cognition therapy
4	Comprehensive application

**Table 8:** Scores in round 2.

Tasks		Mean (SD)	CV^a^
**Oral expression therapy**			
	Read and repeat task: some phrases, including categories of digital, fruit, animals, vegetables, transportation, kitchen supplies, daily necessities, body parts, food, address, location, action, and sports	4.93 (0.26)	0.05
	Read and repeat task: sentences, including proverbs and daily expressions	5.00 (0)	0
	Answering some questions related to everyday life	4.87 (0.35)	0.07
	Naming task: picture naming	5.00 (0)	0
	Naming task: naming list of items with the exact nature	4.93 (0.26)	0.05
	Describing pictures and videos pertaining to daily life	4.67 (0.49)	0.10
**Auditory comprehension therapy**			
	Listening and matching: included digital, color, pictures, and words	5.00 (0)	0
	Yes-no questions	4.93 (0.26)	0.05
	Listening to the passage and answering questions	4.80 (0.41)	0.09
**Cognition therapy**			
	Attention training	4.27 (0.70)	0.16
	Memory training	4.13 (0.74)	0.18
	Reasoning and problem solving	4.20 (0.77)	0.18
**Comprehensive application**			
	Supermarket task: A computer-generated virtual customer purchases goods. The patient has to pick items, count the total amount of money that the virtual customer has selected, and return the change.	4.80 (0.56)	0.12
	Interview task: The patient plays the role of a job seeker. A computer-generated virtual interviewer asks questions about the patient’s personal information (eg, name, gender, age, nationality, education level, birth date, height, weight, specialty, address, and family members) and assesses their language skills by reading text and describing pictures.	4.80 (0.56)	0.12
	Bedroom task: The patient plays the role of a mother. By communicating with the computer-generated virtual daughter, the patient selects clothes for the virtual daughter according to the weather conditions and discusses breakfast and what to choose for dinner and the kind of transportation to use.	4.87 (0.52)	0.11
	Ordering task: The patient acts like a customer to order in a virtual restaurant; communicates with a virtual server synthesized by the computer; requests a certain number of dishes, desserts, cakes, and drinks; and completes the payment.	4.87 (0.52)	0.11
	Park task: By buying tickets, paying, and asking for directions in the virtual ticket office, together with a computer-generated virtual friend, the patient enters a virtual park. The patient and the virtual friend communicate with each other about the scene.	4.53 (0.64)	0.14

^a^CV: coefficient of variation.

#### Usability Testing of the App

**Figure 4:**
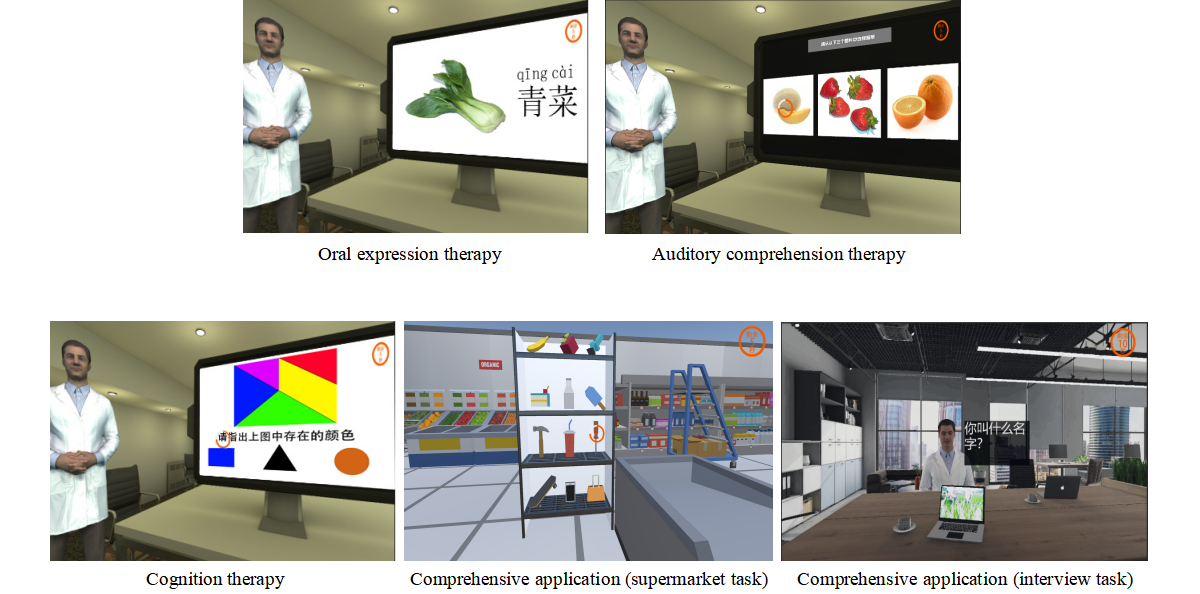
Functionality of the VR-based aphasia therapy app. VR: virtual reality.

All 17 (100%) participants could open the app. A user-centered design philosophy was implemented to create additional interactive and customizable features that indicated a high degree of usability for patients with aphasia. Participants' comments were collected and descriptively analyzed based on the interview outline. The 4 responses were classified based on their feedback. Figure 4 shows the functionality of the VR-based aphasia therapy app.

In response to question 1, “What groups do you think this mobile-based VR speech rehabilitation app is suitable for?”, 12 (70.6%) of 17 participants suggested that the user group could include patients who were discharged or currently hospitalized. Participants stated that with this app, they could correct and refine their language skills, and 5 (29.4%) of 17 participants recommended that the user group should include people, especially those who are under significant financial pressure and do not have easy access to medical treatment but would prefer an opportunity to learn.

In response to question 2, “How does the mobile-based VR speech rehabilitation app differ from face-to-face language training?”, all 17 (100%) participants mentioned the advantages of in-home apps. Participants stated that this app reduces the difficulty of going to the hospital and is more convenient as they do not have to worry about weather and traffic. In addition, 15 (88.2%) of 17 participants reported a high level of immersion and engagement and thought that patients could thoroughly enjoy themselves when using this app without judgment from others. Additionally, there is no need to worry about self-abasement because of the inability to speak fluently. Moreover, 4 (23.5%) of 17 participants critiqued the effectiveness of this app because patients could not receive immediate feedback, and the efficiency of using this app could not be guaranteed.

In response to question 3, “After using the mobile-based VR speech rehabilitation app, what would encourage or hinder your adoption of the app?”, 15 (88.2%) of 17 participants mentioned that the design and arrangement met patients’ daily communication needs; they felt more secure because the training content was designed by a professional rehabilitation team. In addition, 15 (88.2%) participants mentioned that the app was easy to use, and 3 (17.6%) reported low levels of motion sickness.

In response to question 4, “What is your overall rating of the mobile-based VR speech rehabilitation app?”, all 17 (100%) participants indicated that the app was novel, and 5 (29.4%) of 17 participants gave some recommendations, such as considering different training difficulties and increased functionality. All patients expressed interest in language rehabilitation using the new technology.

## Discussion

### Principal Findings

This study developed the first accessible and cost-effective mobile-based VR speech rehabilitation app in China. Through a 2-round Delphi study, panel members reached a consensus on 4 modules and 17 submodules that benefit patients in gaining essential daily communication language skills and various skills in everyday life. Patients get opportunities to interact with the virtual environment and can practice at any time. A review systematically identified and evaluated a series of mobile apps for speech-language therapy and found a lack of interactive and engaging elements in the apps, which failed to make patients self-manage [11]. The content we designed based on VR emphasizes user experience, engagement, and visual appeal to improve patient adherence. We developed real-life scenarios and gaming factors for the VR environment. Patients get opportunities to interact with a virtual environment to practice language in natural context communication settings in VR. We believe that the rehabilitation content will provide patients with a strong basis for returning to society and work.

We developed the content by learning from a model of cognitive communication competence [7]. Language processing is the core of cognition and requires the participation of other nonlinguistic cognitive functions [25,26]. Left hemispheric stroke leading to impairments in language processing often affects other cognitive functions, such as executive function, attention, visuospatial perception, logical thinking, and memory [27,28], which play an essential role in aphasia recovery and rehabilitation [29-31]. Patients' language and nonlinguistic cognitive functions cannot be separated owing to complex neurobiological networks [32]. Nonlinguistic cognitive function in patients with poststroke aphasia influences language rehabilitation outcomes [33].

Language rehabilitation combined with cognitive rehabilitation can improve patients' communication skills [34]. The cognitive domain includes processing, attention, working memory, social cognition, reasoning, and problem-solving [7]. Therefore, to improve the patients’ oral expression abilities, we designed cognitive training pertaining to reasoning, memory, attention, visuospatial perception, and logical thinking. A range of brain regions are related to cognition, and these nonlanguage domains play a role in the ability of patients with chronic aphasia [29].

To emphasize the communication demands of an individual's life, the contextual domain is placed at the top of the cognitive communication competence model [7]. A Cochrane review concluded that communication activity in real-world settings, or functional communication, could improve communicative ability [1]. Communication practices should occur in an ecological context. Therefore, we designed 5 virtual scenario tasks focusing on everyday communication activities and pragmatic conversational skills. Patients may receive language practice opportunities in natural context communication settings in VR. These tasks are a comprehensive practice for the patients, and different short communication dialogues of everyday activities and cognitive exercises were integrated, including oral expression, naming, calculation, attention, memory, and reasoning tasks. VR technology is a promising rehabilitation tool and may be a useful alternative to conventional training [35], but the scene in VR is inaccurate. However, we could create almost everything in VR, and patients experience it as an actual situation, which makes it easy to try new therapeutic strategies. All learning in VR can be transferred to the real world [12]. With clinician shortages and a higher prevalence of aphasia, VR is not meant to replace skilled therapists but to ease clinicians' burdens.

### Limitations

This study had some limitations. First, a range of brain regions are related to attention and executive functioning, and these nonlanguage domains play a role in the abilities of patients with chronic aphasia [29]. Owing to the limitations of intelligent VR, we cannot design some daily instructions (eg, washing clothes, cutting vegetables, cooking). VR could be used to provide an enriched environment where patients could master skills related to daily life that cannot be finished in the hospital. Learning in VR can then be transferred to real-life situations. Second, dialects vary across different areas of China. However, we did not involve dialects from different regions of China to provide options for those who have difficulty understanding and expressing Mandarin. There are nonlanguage abilities that would be worthwhile to consider in aphasia rehabilitation, such as prosody and emotional tone in utterances. However, it is difficult to implement these in the app. In addition, the usability of the app was assessed by only 17 individuals, which may have biased the usability results. In the future, we will design a more significant multicenter trial with a longer-term follow-up. Additionally, this app is meant to improve patients' oral expression abilities, so reading and writing are beyond our consideration. In particular, owing to the current limitations of technology, we cannot plug writing training into VR.

### Conclusion

We conducted a Delphi study and developed a mobile-based VR app. This study constitutes a step toward the development of a combination of health and VR. We believe there is significant potential to make our app an in-home app in the future, contributing to the automation of rehabilitation administration. However, further studies are needed to evaluate the feasibility and efficacy of our app. We hope that our research provides guidelines and references for others in the medical field.

## Abbreviations

ABI: acquired brain injury
AI: artificial intelligence
CV: coefficient of variation
VR: virtual reality
